# Experiences of co‐designing research about a rural Aboriginal well‐being program: Informing practice and policy

**DOI:** 10.1111/ajr.12924

**Published:** 2022-10-17

**Authors:** Lisa Urquhart, Karen Roberts (Dunghutti), Clinton Gibbs (Muruwari), Karin Fisher, Leanne J. Brown, Kerith Duncanson

**Affiliations:** ^1^ Department of Rural Health, College of Health, Medicine and Wellbeing The University of Newcastle Coffs Harbour New South Wales Australia; ^2^ Galambila Aboriginal Health Service Coffs Harbour New South Wales Australia; ^3^ Mid North Coast Local Health District Port Macquarie New South Wales Australia; ^4^ Department of Rural Health, College of Health, Medicine and Wellbeing The University of Newcastle Tamworth New South Wales Australia; ^5^ Hunter Medical Research Institute New Lambton Heights New South Wales Australia; ^6^ Centre of Research Excellence in Digestive Health University of Newcastle Callaghan New South Wales Australia; ^7^ College of Health, Medicine and Wellbeing The University of Newcastle Callaghan New South Wales Australia

**Keywords:** Aboriginal health, collaborative inquiry, critical hermeneutics, indigenous communities, Yarning

## Abstract

**Objective:**

The objective of this study was to explore data and Aboriginal and non‐Aboriginal researchers' experiences and reflexivity in co‐designing research about a rural Aboriginal well‐being program to inform practice and policy.

**Setting:**

Gumbaynggirr, Birpai, Kamilaroi and Awabakal countries located in regional and rural New South Wales, Australia.

**Participants:**

Rural and regionally located research team who co‐designed processes to challenge the status quo about a critically framed, rural‐based Aboriginal well‐being research project.

**Design:**

Researchers drew on data from a research project in an interpretive cycle of collaborative Yarning. Data included 90 published articles, 12 Yarning transcripts and 26 reflective journal text sets, as well as researcher experiences and reflexivity.

**Results:**

The Duguula Gayirray (Yarning together), Yandaarray (walking together) and Duguula Nguraljili (sharing together) co‐design practice model was developed to represent key actions in the context of an Aboriginal well‐being program in a rural context. Actions were supported by seven interpersonal ways of being and were underpinned by respectful relationships between community and researchers.

**Discussion:**

Duguula Gayirray, Yandaarray and Duguula Nguraljili are critical to co‐design practice and are grounded in respectful relationships. Our experiences led us to critique our perceptions of power sharing, equitable partnerships and collaborative knowledges towards opportunity for collective research co‐design.

**Conclusion:**

Duguula Gayirray, Yandaarray and Duguula Nguraljili transformed our understanding of achieving liberation from dominant western research in the context of a rurally located Australian Aboriginal well‐being program. This study contributes to progression of Aboriginal health research practice and policy recommendations, enabling real cultural change in health care with rurally located Aboriginal communities.

**What is already known on this subject:**

- Respecting Aboriginal ways of knowing, doing and learning is essential to research that explores Aboriginal health in rural contexts
- Co‐design of research exploring strengths of a rurally located Aboriginal well‐being program offers an opportunity to challenge Western empirical evaluation measures
- Co‐design approaches are often not clearly described in the literature about First People's health research

**What this paper adds:**

- A collaborative research project about the strengths of an Aboriginal well‐being program provided a platform to explore co‐design in a health research context
- The development of the Duguula Gayirray (Yarning together), Yandaarray (walking together) and Duguula Nguraljili (sharing together) co‐design practice model in the context of a rurally located Australian Aboriginal well‐being program to guide future co‐design health research with Aboriginal communities
- This study is informed by and contributes to the progression of Aboriginal health research policy and practice recommendations, enabling real cultural change in health care with Aboriginal communities in rural contexts

## 1 INTRODUCTION

Aboriginal health projects need Aboriginal community governance to represent holistic, strength‐based complexities and experiences of Aboriginal well‐being.^1^ Privileging Aboriginal ways of knowing, doing and learning when undertaking research exploring Aboriginal health in rural contexts ensures that projects are meaningful and empower Aboriginal people and communities.^1^, ^2^ Aboriginal ways of knowing, doing and learning through Yarning together can affirm relationships that are based on trust, respect and mutual understanding through shared accountability.^3^ Yarning together to co‐design research to explore the strengths of a rurally located Aboriginal group nutrition and exercise program offers an opportunity to challenge disempowering Western empirical evaluation measures and colonial perspectives.^1^

Experiences that enable co‐design in the context of rural Australian Aboriginal and international First Nations Peoples’ health research have been explored in recent literature.^4^, ^5^, ^6^ Reported enablers of co‐design include relational attributes such as community‐based partnerships,^6^ community leadership^6^ and strong trusting relationships.^5^ Reflections on the experience of co‐design also highlight the importance of design flexibility,^5^ learning^5^, ^6^ and acceptability of implementation by community.^4^, ^5^ Dreise and Mazurski^1^ suggest that co‐design is a dynamic and ongoing process that requires joint reflection and reciprocal learning for positive co‐design approaches in Aboriginal contexts.

Co‐design practice is enabled by collaborative and developmental methods that shift ‘power to process’^7^, ^8^ through user‐centred and participatory design to challenge power imbalances in research and health care.^7^, ^8^ In Aboriginal research contexts, co‐design practice relies on the close and ongoing involvement of communities in designing and evaluating meaningful research that embodies respect, liberation and community governance.^1^ Relational Aboriginal knowledge sharing through Yarning^3^ and reflective practices such as Dadirri^4^, ^9^ offer strength‐based ways for community and researchers to research together. Interlinking relational Aboriginal and critical Western research approaches can stimulate meaningful participatory research and positive cross‐cultural co‐design.^10^, ^11^, ^12^ In turn, meaningful co‐design practice can co‐create beneficial First Nations health strategies to foster implementation, dissemination, uptake and sustainability.^4^, ^5^

Despite increasing awareness and enablers, the uptake and exploration of experiences of co‐design practices about First Nation's health research are seldom described in the literature.^13^ There are limited written guidelines or policies to inform rural and remote co‐design best practice within the context of rural Aboriginal nutrition and exercise programs.^14^ The Lowitja Institute's 2020 report ‘Culture is Key: Towards cultural determinants driven health policy’ calls for clearer representation of co‐design to distinguish it from other engagement or consultation processes that inform and influence policy.^14^ Our team has co‐designed a research project centred around a rurally located, Aboriginal nutrition and exercise program that has informed a co‐design practice model. The objective of this article is to interpret data and Aboriginal and non‐Aboriginal researcher's experiences and reflexivity about co‐design in the context of a rural Aboriginal nutrition and exercise program to inform practice and policy.

## 2 METHODS

### 2.1 Background

Spring into Shape is an Aboriginal group nutrition and exercise program, described by program participants as a well‐being program (hereon referred to as a well‐being program), that has run for 17 years in Gumbaynggirr Country on the Mid North Coast of New South Wales, Australia. The well‐being program was co‐designed by the community, Aboriginal Health Service and Mid North Coast Local Health District. Spring into Shape is supported by KR as the program coordinator and LU as the dietitian. Over the past 5 years, KR and LU have developed a relationship with program participants and have worked together with community to design a research project^10^ to investigate the strengths of the Spring into Shape program. The overall research project has evolved to include a co‐designed literature review,^15^ and current Yarning project (to be published separately).

**FIGURE 1:**
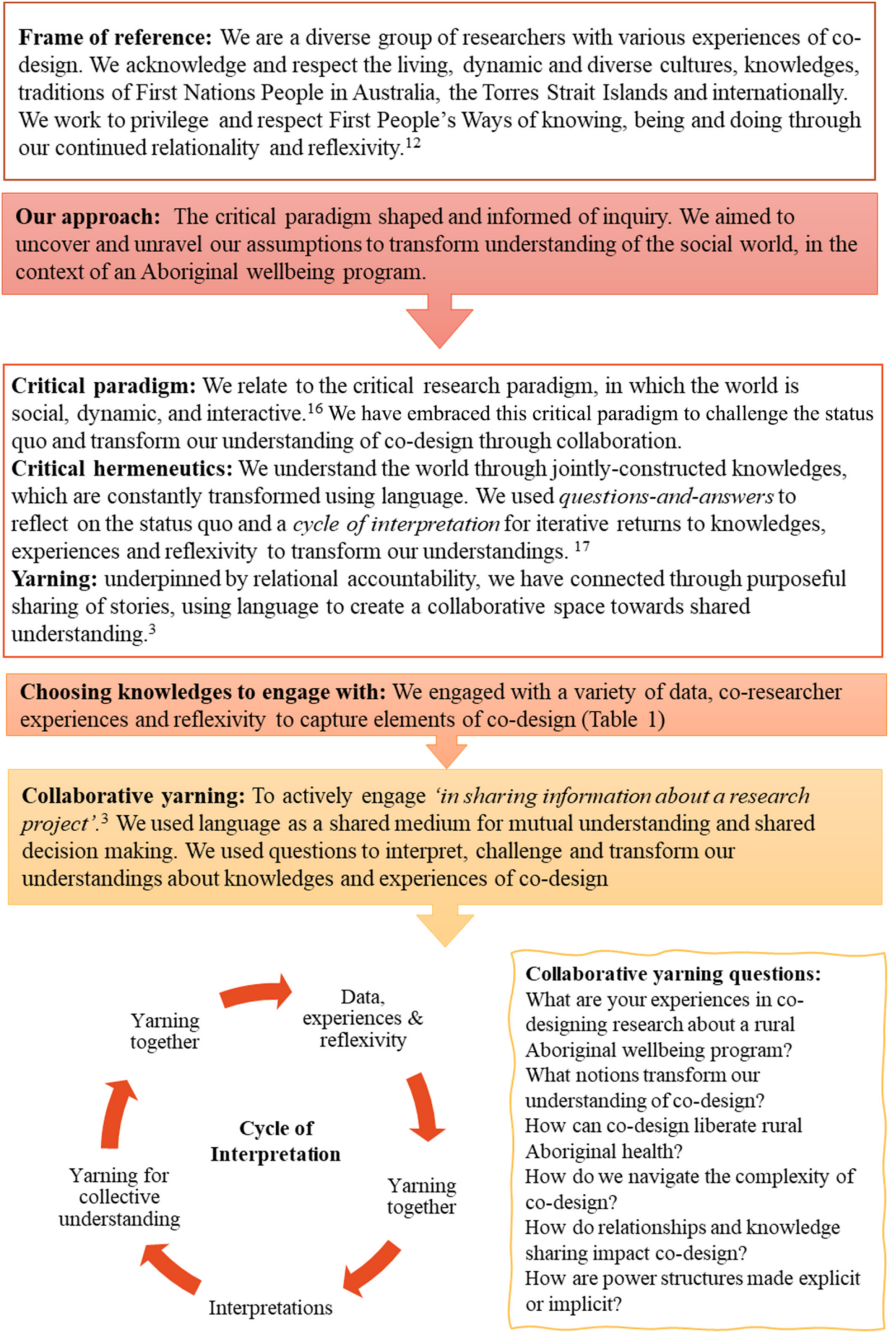
Frame of reference and approach we used to interpret data, experiences and reflexivity about co‐design

The literature review was informed by critical hermeneutics and collaborative Yarning (see Figure 1 for definitions). Three Aboriginal (including KR and CG) and four non‐Aboriginal researchers (LU, KF, LB, KD) completed the review.^15^ For the review, researchers interpreted texts to transform understandings about how success is described in literature relating to First Nations People's nutrition and exercise programs. Throughout the review,^15^ researchers noticed how elements of success intertwined with participatory approaches. Researchers were inspired to revisit those same texts to inform our understanding and interpretations of co‐design for this article. The Yarning project about the strengths of ‘Spring into Shape’ involves one Aboriginal (KR) and non‐Aboriginal (LU) researcher Yarning with individual participants and stakeholders about the group well‐being program. Again, researchers noticed that notions of co‐design were emerging from Yarning about Spring into Shape and revisited these transcripts to elicit elements of co‐design.

#### 2.1.1 Participants and setting

Six rural and regionally located researchers who have previously worked together formed a research team. Two researchers are Aboriginal (KR, CG) and four researchers are non‐Aboriginal (LU, KF, LB, KD). Researchers are located on Gumbaynggirr Country (KR, LU) (Coffs Harbour), Birpai Country (CG) (Port Macquarie), Kamilaroi Country (KF, LB) (Tamworth) and Awabakal Country (KD) (Newcastle), New South Wales, Australia. Researcher backgrounds are Aboriginal Health Worker and Aboriginal Elder (KR), project manager (CG), dietitian and PhD student (LU), dietitian and researcher (LB, KD) and nurse and researcher (KF). ‘Spring into Shape’ program participants contributed knowledges towards this co‐design component of the overall research project.

### 2.2 Research approach

To interpret co‐design elements, researchers collaboratively decided to use a critical hermeneutic methodology^16^ and Yarning methods^3^ as they were familiar, corresponded to our frame of reference and best positioned to achieve the research aim (see Figure 1).^17^ We used data alongside researcher experiences and reflexivity to explore co‐design. Data included 90 published articles, 12 Yarning transcripts and 26 reflective journal texts. Researcher experiences and reflexivity added further depth to interpretation (see Figure 1). A snapshot of the co‐design data, experiences and reflexivity are outlined in Table 1. Data, experiences and reflexivity informed our interpretation of co‐design.

**TABLE 1:** Knowledges, experiences and reflexivity components used to inform researcher interpretation of co‐design elements

Contributors to knowing, doing and being:		Examples of activities
Data		Yarning interview transcripts Collaborative and individual written reflections Literature review texts (published articles)
Experiences	Community based	Community events, e.g., Elder's Olympics and National Aborigines and Islanders Day Observance Committee week community day Weekly involvement in well‐being group activities and meeting and Yarning with the Aboriginal Health Service staff and clients Meetings and Yarning with Elder's group
	Research process	Process of co‐developing ethics application and designing graphics for recruitment of participants to the research Doctoral research confirmation presentation and process—diagrams and artworks Critical hermeneutic collaborative inquiry informed literature review Planning the collaborative Yarning and visual interpretation of well‐being program Meetings and Yarning with Spring into Shape participants, staff, and stakeholders ‘Asking back’ diagrams and representations of Yarning (methods paper, literature review, three conference presentations and confirmation) Research Yarning with research participants Cultural mentoring with local Elder Research and health capacity building activities with staff
Reflexivity	Collaborative	Yarning together—social, research topic, collaborative and therapeutic Yarning Ethics application and amendment processes (accountability and reporting back to Aboriginal Health and Medical Research Council ethics) Conference presentations (including responses to audience questions) Publication submission (manuscript preparation and reviewer responses) Research timeline pauses, adjustments and disruptions
	Individual	Individual thoughts and changes in practice Responsibility and accountability to the research, community and researchers Doctoral research confirmation document (including defence and feedback)

Researchers engaged in nine meetings which were 60–90 min in duration. Researchers used a *question‐and‐answer* approach to Yarn about co‐design using data, experiences and reflexivity (Table 1). Yarning partners agreed on the agenda, time and frequency of meetings. Meetings were conducted and recorded via Zoom (San Jose, CA: Zoom Video Communications Inc). Prior to each meeting, LU listened back to the recordings to draw out and iteratively progress themes and interpretations. Each meeting involved Yarning about our relationships with the data, experiences, reflexivity, as well as themes and questions that evolved from our Yarning. We also explored individual‐level differences and similarities in our interpretations.

Data were managed in NVivo (Version 12.4, QSR International Pty Ltd). LU used NVivo to code text segments which answered the Yarning questions, or which sparked new questions for further Yarning by researchers.

**FIGURE 2:**
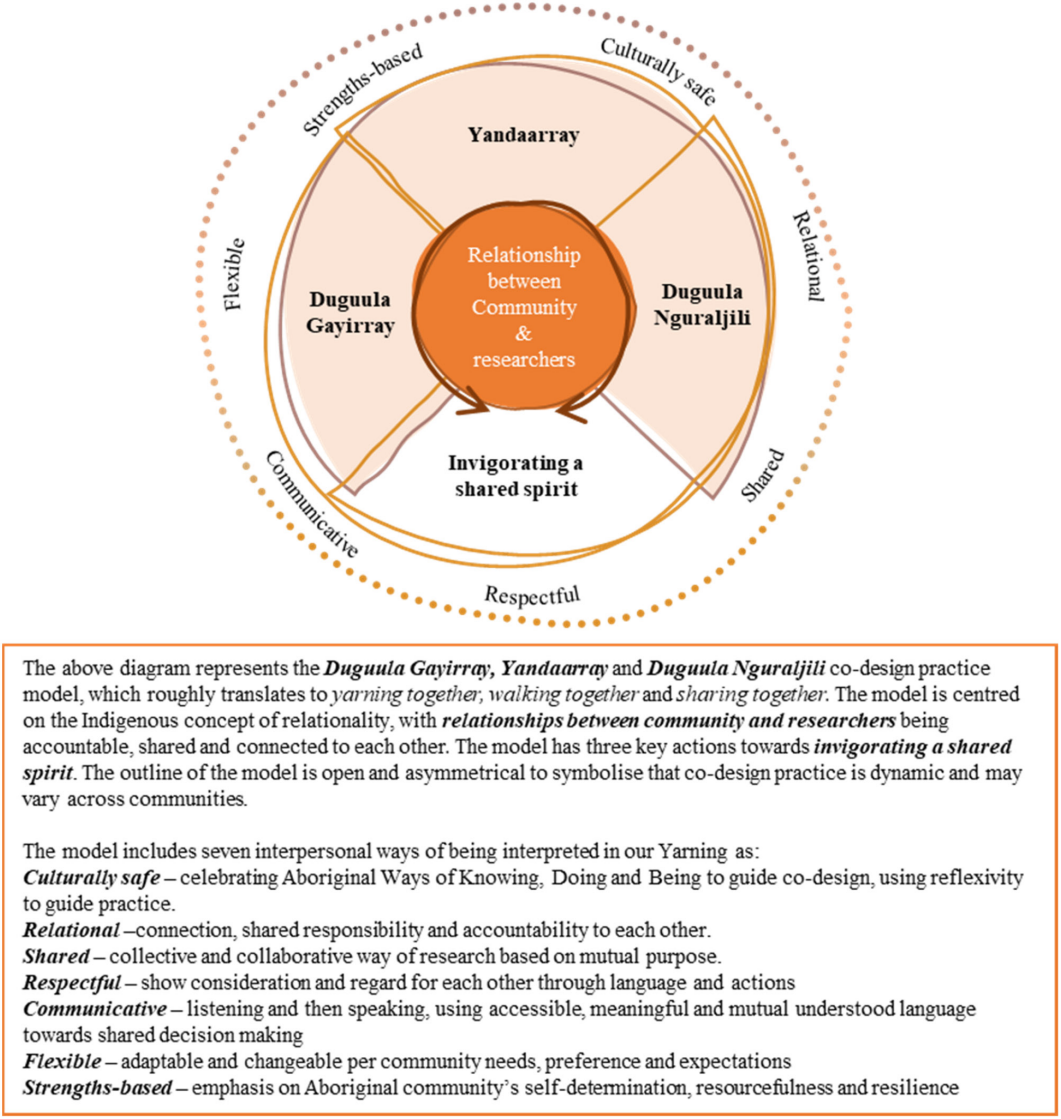
Representation of results from collaborative Yarning and critical hermeneutic process

Researchers checked and refined initial interpretations through further collaborative Yarning. KR and LU yarned with program participants and Aboriginal Health Service Staff to check interpretations. A model was developed by researchers that articulates important elements for co‐design practice in the setting of a rurally located Aboriginal well‐being program (see Figure 2 in Section 3).

### 2.3 Ethics

This research project has ethical approval from the Aboriginal Health and Medical Research Council Ethics Committee, NSW (1411/18) and University of Newcastle Ethics Committee, NSW Australia (H‐2018‐0238). Researchers applied COREQ (COnsolidated criteria for REporting Qualitative research) checklist to the article.^18^

## 3 RESULTS

A collaborative research project about the strengths of the Spring into Shape Aboriginal well‐being program provided a platform from which to explore co‐design in the health research context. Collective interpretations of data, experiences and reflexivity about co‐design practices transformed researchers' understanding of power, authority and dominance. Key concepts from interpretations were collaboratively explored with knowledge holders of Gumbaynggirr language and local Elders to articulate the complex meanings of co‐design. From this exploration, we conceptualised the Duguula Gayirray, Yandaarray and Duguula Nguraljili co‐design practice model, which roughly translates in English as Yarning together, walking together and sharing together (see Figure 2). The model is underpinned by the Indigenous concept of relationality, where relationships between researchers, Aboriginal community members and leaders hold accountability, and connection to each other and to the research (Figure 2).^12^ Key co‐design actions in this research about a rurally located Aboriginal well‐being program were Yarning together to make power dynamics transparent, walking together to navigate complexity and sharing together to employ diverse knowledges. Co‐design which encompasses Yarning, walking and sharing together gives opportunity for invigorating a shared spirit. The interpersonal ways of being that supported co‐design elements are depicted in the outer section of Figure 2. The characteristics that researchers described as encapsulating co‐design health research practice were strength‐based, culturally safe, relational, flexible, communicative, respectful and shared.

### 3.1 Duguula Gayirray (Yarning together) to make power dynamics transparent

Collective interpretations made explicit that Yarning together to make power dynamics transparent was an essential element of the co‐design practice model (Figure 2). We understood that power dynamics are the often‐implicit ways power affects relationships between people. In Australia, power dynamics are distorted due to the domination of Aboriginal people through the violence of colonisation, subjugating Aboriginal knowledges, voices, lands, languages and cultural structures. We noticed that power dynamics were always present in co‐design relationships, and it resonated with us to work to make power dynamics transparent by Yarning together. The action of making power dynamics transparent was enabled by relational respect and trust, in each other and to the research process.

The notion of representative voices helped to make power dynamics transparent. There are subtleties in how voices were represented (or were considered to have been represented) in the co‐design process. For example, we examined the implicit power dynamics in community engagement actions of ‘consultation’ and ‘collaboration’ which implied the representation of community voices in research. We noticed that ‘consultation’ usually involved a one‐way communication from researcher to community, without ongoing representation of multiple voices. However, ‘collaboration’ meant ‘working with’ and Yarning together, in a back‐and‐forward motion to develop a shared understanding using language. With representative voices Yarning together, collaboration enhanced opportunity to reflect on language use (or misuse) for research actions to reveal power dynamics.

Yarning together using language that was clear, understandable and meaningful improved the transparency of power dynamics. Using Aboriginal languages,^19^ Aboriginal English^20^ and English,^21^ appropriately and in a culturally safe way fostered transparency and inclusion in the co‐design process. We reflected that the dominance of English through the act of colonisation risks diminishing the rich, relational and complex meanings of Aboriginal languages. Instead, inclusive choices of appropriate language, that may or may not include English, required listening and collaboration to clarify, reflect and ask questions. By promoting understanding through accessible language, each person involved could interpret and critique power dynamics to enable transparency (see quote below). Underlying inclusive and appropriate language was a sharing and trustworthy relationship between community members and researchers.

…open and transparent communication could occur ensuring that the same message was being delivered to all stakeholders throughout the whole process.(Literature data, A diabetes support group for Nywaigi women, Australia^22^)

Yarning together revealed opportunities to enhance co‐design transparency through formal and informal processes (see Table S1). Formal processes included collaborative memorandums of understanding,^23^ shared ethics applications and written agreements between Aboriginal community‐controlled health organisations and other parties. Informal processes enhancing transparency of power dynamics included researchers attending community events,^24^ Yarning with community Elders, and developing respectful partnerships between community health providers and university academics.^5^ Transparent formal and informal research processes which supported co‐design were based on community needs and established community practices.

By Yarning together, co‐designers can work to reveal power dynamics between rurally located communities and institutions. For co‐design, institutional researchers need to be present, available and open to co‐construct research with rurally located Aboriginal communities. For example, rural Aboriginal communities may prefer face‐to‐face and co‐located Yarning about co‐design compared to video or telephone conferencing. Thus, researchers must not presume using technology is the most effective and efficient way of working. The quote below, from a literature review text, shows how researchers worked to establish more transparent power dynamics through communication, co‐location and a memorandum of understanding for co‐design, and was applied to our project:

… this involved many months of both face to face and by telephone meetings with consensus decision‐making processes and, at an organisational level, the signing of a Memorandum of Understanding.(Literature data, Ngāti and healthy, Aotearoa New Zealand^23^)

Yarning together to collaboratively establish appropriate language, processes and privilege community voices may work to expose and critique power dynamics towards the possibility of equitable power sharing in co‐design.

### 3.2 Yandaarray (walking together) to navigate complexity

Another element of the co‐design practice model in the context of a rurally located Aboriginal well‐being program was walking together to navigate complexity (Figure 2). Western knowledge processes aim to simplify questions and answers by reducing variables. Instead, we yarned that co‐design was complex and to find solutions, we needed to walk together to navigate complexities, as community and researchers. We interpreted walking together as a symbolic expression of community and researchers journeying, side by side, in a trusting relationship to co‐design research. We explored the enablers of time, space, relationships and self in the co‐design journey.

Navigating complexities by walking together required time to develop relationships and trust. Processes in co‐design were not always concordant to institutional and funding time‐linear deadlines. We learned from our experiences and the data that walking together reduced the risk of overlooking complex time‐dependent values and priorities, as described in quote below:

Such relationships and trust take time and there needs to be sufficient co‐design/participatory process to establish the relationship.(Literature data, Aotearoa New Zealand^5^)

In many Aboriginal communities, time is a nonlinear concept with no beginning and end and no direct path to follow. Hence, the co‐design journey is dynamic and in constant flux with ongoing changes and improvements based on community need (see quote below). The complexity of the fluidity, flexibility and cyclical nature of co‐design required walking together towards a shared process.

…flexibility is imperative, so there are no set rules or formal structure in terms of timing.(Literature data, Waminda's Wellbeing Program, Australia^25^)

Navigating the complexity of spaces together was explored as another co‐design enabler. Co‐design was supported by walking together ‘in community’, ‘on Country’ and with others. These spaces were considered to be culturally safe and familiar to community members. Our Yarning reflected on the difficulties of connecting and building relationships in virtual spaces (e.g. videoconferencing) especially through the COVID‐19 pandemic. It was important for researchers to explore spaces specific to each community as outlined in the below reflection:

…at our [rural] site, relationships were much stronger in‐person [rather than virtual], people wanted to connect face‐to‐face. This was different to metro, who met virtually…(Research experience, Aboriginal researcher, co‐design Yarning meeting 1)

Collaboration and trust between community and researchers is essential to co‐design relationships (Figure 2). Co‐design relationships needed to navigate relational complexities such as community connections, staffing changes and interpersonal tensions. Relational complexities often relied on Aboriginal community researchers walking with external researchers to instigate, navigate and build community relationships together. For Aboriginal community researchers, building relationships required using their lived knowledge about their community, and, their time, effort and accountability to both their community and researchers.

Navigating complexity of ‘the self’ enabled community and researchers to walk together. Understanding ‘the self’ required introspection and reflexivity as the individual, to draw out motivations and expectations. In our Yarns, we connected with Dadirri, reflective principles which tap into the deep spring within us, from the Ngan'gikurunggurr and Ngen'giwumirri languages of the Daly River region.^9^ Dadirri is a quality of *inner, deep listening and quiet, still awareness*.^9^ Dadirri offers community and researchers a tool to become external observers of their own actions, which facilitates a shift towards shared understanding.

We noticed that the complexities of co‐design practice interlinked and often needed simultaneous navigation. For example, the temporal notion of deadlines, spatial notions of rural distances and relational aspects of staff turnover can disrupt processes, if not carefully navigated.^23^, ^26^ The interlinking of complexities is embodied in the below quote:

The establishment of a good collaborative research team with a shared focus, and the community consultation took time, with the cost being the loss of the opportunity to secure funding from a national source.(Literature data, Ngāti and healthy, Aotearoa New Zealand^23^)

Walking together to navigate complexity is a key opportunity for equitable partnerships in co‐design. It requires flexibility, attention to and investment in time, space, relationships and the self (see Table S1).

### 3.3 Duguula Nguraljili (sharing together) to employ diverse knowledges

Sharing together to employ diverse knowledges was another element for the co‐design practice model (Figure 2). Dominant Western knowledge diminishes opportunity for many ways of knowing and learning. Instead, by recognising, valuing and employing the rich and varied ways of knowledge sharing, community and researchers can build positive co‐design practice. Diverse ways of knowledge sharing were employed by taking time to share, and working to build relationships. For transformative co‐design, diverse knowledge sharing needs to be a reciprocal practice, with community and through oral ways (Yarning).

Our program combines new learning with traditional knowledges. I learn from you, and you learn from me, that's how it's got to be.(Collaborative reflexivity, Aboriginal researcher, literature review meeting 3)

Privileging knowledge that comes from a local, community perspective enables research co‐design.^21^, ^27^ Community knowledge is rich in the culture, history and complexity of local people. Local voices provide strength‐based knowledge, which guides the co‐construction of research that is tailored for local community. Researchers must recognise that the knowledge shared by community is shared in good faith and with intention, and some knowledges are not shared with deliberateness. By honouring and co‐exploring shared knowledges, co‐designers can build trusting relationships (see below quote). By privileging local wisdoms, researchers respect and engage the diversity of Aboriginal community expertise.

We need to acknowledge that Aboriginal knowledge is not lost, it's about revitalising, acknowledging and incorporating into programs.(Collaborative reflexivity, Aboriginal researcher, literature review meeting 2)

Sharing together employed different knowledge types for co‐design practice.^28^ We understood that no one type of knowledge can dominate the co‐design process. Knowledge types that support co‐design practice include cultural, community, technical, creative, spiritual, scientific and organisational wisdoms. By sharing together, community and researchers bring different knowledge types to support co‐design practice.

The ways of sharing knowledge for co‐design practice are as diverse as the types of knowledge. We recognised that knowledge sharing extends far beyond written methods to include oral stories, artefacts, painting, poetry, song and other unwritten knowledges and experiences.^29^, ^30^ For us, it was the unwritten ways of knowledge sharing that accessed diverse and constructive ways for co‐design practice. For example, the research team needed to transform a predominantly reading and writing‐based literature review processes to a verbal format so that the process was accessible and comfortable for all contributors (see Table S1). Sharing together for diverse knowledges is epitomised by sitting and Yarning in community, listening, reflecting and co‐constructing a research project, demonstrated in the below quote.

That's the way we start to understand the research, explaining it in that way…we sit and talk…we share that moment together.(Research experience, Aboriginal researcher, co‐design Yarning meeting 7)

We noticed that nonlinear knowledge sharing is a meandering journey, guided by community, which supports co‐design practice. This type of sharing includes ways of being and doing that are flexible, build on strengths and are respectful through cautious culturally safe practice (see quote below). The nonlinear knowledge sharing journey co‐explores learning needs of those involved. It does not assume the learning needs, pace or direction of community or researchers. Instead, it shows vulnerability and genuine interest by respectfully asking to learn and share knowledge. Nonlinear knowledge sharing employs diverse ways of knowing:

Well that's the thing about Aboriginal culture. We really see learning as being cyclic. It goes around. You never stop learning and you're learning from younger ones and older ones and we're passing information along and Spring into Shape is just one of those programs that really lends itself to that sharing and caring.(Yarning Data, Y11)

By sharing together in co‐design, we reflected that researchers and community can celebrate and engage collaborative ways of knowing to challenge the status quo of dominant knowledge. Sharing together to employ diverse knowledges involves the characteristics of flexibility and respect and the conditions of co‐constructed, reciprocal, nonlinear and privileged local knowledges.

### 3.4 Invigorating a shared spirit

The notion of invigorating a shared spirit was the culmination of Yarning, walking and sharing together in the co‐design model (Figure 2). Invigorating a shared spirit intentionally celebrates Aboriginal culture to strengthen co‐design practice. It is a spirit that fosters excitement and passion for the future of co‐design for rurally located Aboriginal well‐being program research. Invigoration of a shared spirit provides opportunity to unsettle the status quo of research and health, while enhancing research rigour through Aboriginal ways of knowing, being and doing. Invigorating a shared spirit is enabled through conscious communication (Yarning together) towards power sharing (see quote below), reflexivity and flexibility (walking together) towards equitable partnerships, and appropriate research methods (sharing together) towards collaborative knowledges, cultivating in collective co‐design about Aboriginal health and research.

Australia might be at the cusp of changing language about research with an Aboriginal community rather than doing research on the community.(Written reflection data, Aboriginal researcher, literature review meeting 2)

Invigorating a shared spirit towards the possibility of power‐sharing relationships requires all stakeholders to use conscious communication. Conscious communication enables an invigorated spirit through deep listening and Yarning to empower one another. Stakeholders can hear the needs of others when communication is free of coercion and motivated towards mutual understanding and shared decision‐making. Through conscious communication, Aboriginal community members and researchers can begin to explore how their histories, knowledges and actions can expose and unsettle the dominant research dialogue towards power‐sharing relationships:

With this basis for accountability established, open and transparent communication could occur ensuring that the same message was being delivered to all stakeholders throughout the whole process. A mutual understanding of these factors between all participants from the outset enabled all invited health professionals the opportunity to work closely and respectfully with and within this Indigenous community.(Literature data, A diabetes support group for Nywaigi women, Australia^22^)

Invigorating a shared spirit offers freedom to be changeable through reflexivity. Through self and collaborative reflexivity, researchers and community can carefully think about their motivations, histories and culture to shape and transform the co‐design of research (see quote below). This involves walking together to co‐design research that is flexible, changeable and based on equitable partnerships. Flexibility offers a freedom from structured, selective scientific study towards inclusive, perceptive and pragmatic research which changes as people change.

Understand and know where community are coming from – we are careful not to re‐traumatise. This comes from being in community and understanding community ‐ trying to connect with people, think about actions and build trust.(Written reflection data, non‐Aboriginal researcher, literature review meeting 3)

Invigorating a shared spirit challenges the status quo of the dominant research paradigm to enable co‐design practice using collaborative knowledges. By applying Aboriginal research methods, such as Yarning and artworks, underpinned by relationality, an invigorated spirit celebrates knowledge beyond Western science. By sharing together, experienced Aboriginal and non‐Aboriginal researchers can align Aboriginal and Western methods for cultural validity and enhanced rigour in the co‐design process. Rigorous co‐design and collaborative knowledges strengthen the research process and ensures the application of appropriate methods to answer the co‐created research question.

Finally, invigorating a shared spirit through Yarning, walking and sharing together cultivates collective co‐design. Collective co‐design is possible when community and researchers make deliberate choices towards the emancipation of Aboriginal people, together. Their intentions, language and actions must celebrate Aboriginal culture, sovereignty of Aboriginal knowledges and be supported by interpersonal co‐design ways of being (see Figure 2). A collective co‐designed future authenticates power‐sharing, collaborative ways of knowing and equitable partnerships through Yarning, walking and sharing together.

## 4 DISCUSSION

The process of critical hermeneutic collaborative Yarning about our co‐design data, experiences and reflexivity led us to critique and challenge our perspectives, creating depth to our individual and collective horizons of understanding. To our knowledge, this is the first research project to develop a co‐design practice model in the context of a rurally located Aboriginal well‐being program. The co‐design practice model is centred on relationships between community and researchers, with three key actions towards invigorating a shared spirit, and seven interpersonal ways of being. The outline of the model is open and asymmetrical to symbolise that co‐design practice is dynamic and diverse across heterogenous, rurally located communities.^2^ The Duguula Gayirray, Yandaarray and Duguula Nguraljili co‐design practice model aligns with the Indigenous concept of relationality, through a shared responsibility and accountability to each other, community and the research.^12^

The results of our interpretive process align with policy literature for co‐design research towards tangible benefits for Aboriginal communities.^14^, ^31^, ^32^, ^33^, ^34^ Within a rurally located, Aboriginal well‐being research context, co‐design counters distrust in policy and health care research through open and collaborative processes, ensuring that projects are meaningful and empower community.^1^, ^31^ Consistent with policy innovations such as the Uluru Statement from the Heart, foundations of co‐design in research can help redeem trust deficits.^35^

Power‐sharing relationships can transform health and research in rural Aboriginal communities.^36^ The results of our co‐design interpretation show a need to make power dynamics transparent by Yarning together for the possibility of power sharing. This notion is supported by Dillon^31^ who stipulates that power imbalances must be recognised and denounced by individuals, community and institutions as they undermine effective co‐design and collaboration. For Aboriginal communities who are rurally located, collaboration may include co‐navigating expansive distances to Yarn together, co‐exploring representative voices through accessible language(s) and co‐building processes to safeguard community representation.

This work contributes to further understanding about how walking together to navigate complexities can foster equitable partnerships which are based on trust, mutual responsibility and ethics.^33^ Aboriginal people and cultures in rural locations are distinct and varied, so equitable partnerships need to be place‐based, trusting relationships which have developed over time.^31^ In co‐designed research, equitable partnerships can work towards strength‐based, positive outcomes which meet Aboriginal People's and communities' needs and expectations, and celebrate Aboriginal culture, expertise, capabilities and values.^6^, ^33^

Our results show that collaborative knowledges can be privileged through sharing together to employ diverse knowledges. Supporting collaborative knowledges is a conscious action by communities and researchers to shift the process of knowledge creation and translation that occurs with communities.^37^ This shift facilitates community governance through local wisdoms, for the co‐design process.^34^, ^38^ Collaborative knowledges in co‐design need to advocate for Aboriginal data sovereignty. Data sovereignty is a global movement concerned with the right of Indigenous peoples to govern the creation, collection, ownership and application of their data.^34^ Ultimately, collaborative knowledges that work to privilege Aboriginal data sovereignty, local community wisdoms and beliefs can govern and produce data which are meaningful and relevant to local community needs.^33^, ^34^

It is important to acknowledge that research and policy co‐design and participatory rhetoric remain polysemic, meaning many interpretations are possible.^31^ Aboriginal communities in rural locations are tasked with an ongoing need to critically interpret and question co‐design research proposals and practice, with limited available guidance on ethically based research design.^32^, ^33^ Communities are still required to ask and monitor research design questions about who is included and excluded, who benefits and how power is distributed.^31^ There are ongoing risks of co‐design for communities which include responsibility shifts, failing accountability, loss of democracy, re‐enforced inequalities and implicit demands.^31^, ^39^ Further collaborative research which strengthens transformative co‐design policy, practice and monitoring will need to move past the buzzwords and rhetoric and strive to create meaningful change in research practice (see Table S1).^5^, ^38^, ^40^ Future co‐design practice, in our research and that planned by others, could be extended to include community members in a critical hermeneutic process to further transform understandings of co‐design practice.

The Duguula Gayirray, Yandaarray and Duguula Nguraljili co‐design practice model will be used by community and researchers to guide co‐design practice for further collaborative Yarning as part of an ongoing research project on Gumbaynggirr Country. The next steps will involve a group of Spring into Shape participants and researchers joining as researchers to represent their shared interpretations of Spring into Shape by Yarning, walking and sharing together while co‐creating an artwork. As a group of Aboriginal and non‐Aboriginal researchers, we anticipate that sharing our model will support other rurally located Aboriginal communities and researchers co‐design practices for health and well‐being research.

We anticipate this co‐design practice model will assist to inform Aboriginal well‐being co‐design policy. For example, by Yarning together and sharing together, community and researchers can celebrate diverse knowledges through collective understanding using language. Diverse knowledges may link cosmos, spiritual and land systems with oral, artistic or music artefacts and science knowledges in a complex relational connection.^12^ Through collectively understanding how diverse knowledges are interwoven in research, co‐designers can challenge dominant western and policy thinking to transform how research is recognised, valued and shared (see Table S1 for examples of how the model may be applied to and extend existing policy).

## 5 CONCLUSION

Researchers used collaborative Yarning with critical hermeneutics to develop the Duguula, Yandaarray and Duguula Nguraljili co‐design practice model based on data, experiences and reflexivities. The co‐design practice model comprises of three key actions towards invigorating a shared spirit and seven interpersonal characteristics that are the foundations of co‐design practice. Respectful relationships between community and researchers are critical to transparent, diverse, complex, communicative and shared co‐design practice. Duguula Gayirray, Yandaarray and Duguula Nguraljili transformed our understanding of co‐design achieving liberation from dominant Western research in the context of a rurally located Australian Aboriginal well‐being program. This study is informed by and contributes to progression of Aboriginal health research practice and policy recommendations, enabling real cultural change in health care with rural Aboriginal communities.

## AUTHOR CONTRIBUTIONS

LU: conceptualization; data curation; formal analysis; investigation; methodology; project administration; writing – original draft; writing – review and editing. KR(D): conceptualization; formal analysis; investigation; methodology; supervision; writing – review and editing. CG(M): conceptualization; formal analysis; investigation; writing – review and editing. KAF: conceptualization; formal analysis; methodology; supervision; writing – review and editing. LJB: conceptualization; formal analysis; methodology; supervision; writing – review and editing. KD: conceptualization; formal analysis; methodology; supervision; writing – review and editing.

## FUNDING INFOMATION

The author(s) disclosed receipt of the following financial support for the research, authorship and/or publication of this article: Research Training Program Student Support Allocation, School of Health Sciences, College of Health, Medicine and Wellbeing, University of Newcastle.

## CONFLICT OF INTEREST

The author(s) declared no potential conflicts of interest with respect to the research, authorship and/or publication of this article.

## ETHICAL APPROVAL

This research project has ethical approval from the Aboriginal Health and Medical Research Council Ethics Committee, NSW (1411/18) and University of Newcastle Ethics Committee, NSW Australia (H‐2018‐0238).

## Supporting information


Table S1
