# Supplementary material for: Experiences of co‐designing research about a rural Aboriginal well‐being program: Informing practice and policy
Source: Aust J Rural Health. 2022 Oct 17;30(6):747–59. doi: 10.1111/ajr.12924 (PMC10092418; doi:10.1111/ajr.12924)
Supplement: Supplementary file 1 — Table S1 [file AJR-30-747-s001.docx]

**Table S1: Co-designing Aboriginal health research - mapping practice to policy**

| Policy reference informing our co-designed research | Our planned research activity | Our actual research activity | Co-reflections | Relation to model | Model highlighting principles and actions for community, researchers and/or policymakers |
| --- | --- | --- | --- | --- | --- |
| NH&MRC Keeping Research on Track II^1^   - Levels of Aboriginal participation - Aboriginal people skills development - Meaningful research methods - Acknowledge contributions. | Literature review with AHS co-researchers. Planned to reading and responding to articles and grey literature. Deemed not feasible as method did not fit with diverse knowledges and ways of understanding. | Yarning about literature using quotes and themes as topics for discussion rather than whole articles which allowed yarning to flow freely for transformation of understanding. | Staffing and timeline changes meant the original plan was not feasible or appropriate.  Yarning that drew on co-researcher rich life experiences informed a transformative literature interpretation. Diverse local knowledges were central to informing results. | Yandaarray (Walking together) to navigate complexities – flexible, strengths-based, relational.  Duguula Nguraljili (Sharing together) to employ diverse knowledges – culturally safe, respectful, shared, strengths-based  Duguula Gayirray (Yarning together) to make power dynamics transparent – communicative, respectful, culturally safe. | Aboriginal health co-designed research needs to be flexible.  Explicit need to make methods suitable for individuals and groups, consider communication differences and travel distances in rural settings.  Revise contributor guidelines for publication to explicitly acknowledge different types of knowledges e.g., cultural  Revise author requirements for identification of First Nations People on an author list |
| Ethical conduct in research with Aboriginal and Torres Strait Islander Peoples and communities: Guidelines for researchers and stakeholders ^2^   - Actively engaging participants and communities in negotiations about the meanings of the research topic and the methods of research. | Ongoing collaborative meetings and genuine community engagement about the research. Formal and informal meetings with AHS^†^ manager, program coordinators, local Elder’s group and program participants. | Co-researchers invited to participate and assist at community events – i.e., NAIDOC^‡^ week, Elder’s Christmas party, Elder’s Olympics. Invited to collaborate with AHS staff in other areas. Development of social and collegial relationships. | Relationships form the basis for engaging with community. Research forms part of community negotiations and discussions, along with social, therapeutic and collaborative yarning. | Relationship between Community and co-researchers  Yandaarray (Walking together) to navigate complexities – flexible, strengths-based, relational.  Invigorating a shared spirit. | Co-designed research and community are relational and cannot be separated.  Reflexively revise expectations of timing of research to ensure time to build relationships. For example – apply principles of Dadirri.^3^  Develop reporting requirements about collaboration and communication beyond ‘research’ activities. |
| Closing the Gap ^4^   - Driving their own development by making evidence-based decisions on the design, implementation and evaluation of policies and programs for their communities to develop local solutions for local issues. | Community and AHS and Local health service co-designed program delivered for 17 years. Co-designed research project to understand success of local program. | Co-researchers invited by AHS to explore success of long-standing program. Elders and cultural mentor driving knowledge development about local program. | Community knowledges in partnership with AHS have successfully built and maintained wellbeing program that benefits community. | Duguula Nguraljili (Sharing together) to employ diverse knowledges – culturally safe, respectful, shared, strengths-based  Duguula Gayirray (Yarning together) to make power dynamics transparent – communicative, respectful, culturally safe.  Yandaarray (Walking together) to navigate complexities – flexible, strengths-based, relational. | Revise acknowledgment and remittance of expertise and evidence-base which does not fit within dominant ways of knowing.  Strengthen longer term funding commitments to support successful rural programs to continue and grow. |
| NSW Aboriginal  Health Plan  2013-2023^5^   - Strategic Direction 6: Strengthening performance monitoring, management and accountability | Co-researchers develop research agreement between AHS and University as part of ethics submission. The agreement outlined how the project would be undertaken and monitored to ensure accountability. | Co-researchers complete monitoring requirements outlined in the research agreement. Monitoring and accountability extended through community accountability e.g., ‘showing up’, being present and yarning about how the research will work, changing processes to work for community. | Our accountability extended beyond institutional structures and involved many community members through yarning and building two-way understanding to co-create a research project to support local Aboriginal health. | Duguula Nguraljili (Sharing together) to employ diverse knowledges – culturally safe, respectful, shared, strengths-based  Duguula Gayirray (Yarning together) to make power dynamics transparent – communicative, respectful, culturally safe.  Yandaarray (Walking together) to navigate complexities – flexible, strengths-based, relational. | Revise accountability frameworks to ensure rural community voice is represented beyond institutional structures.  Ensure a wider representation of accountability (local rural Aboriginal community processes and Western formalised) is incorporated by co-researchers into monitoring, management of research. |
| †Aboriginal Health Service  ‡National Aborigines and Islanders Day Observance Committee. | | | | | |

1. National Health and Medical Research Council. Keeping research on track II : A companion document to Ethical conduct in research with Aboriginal and Torres Strait Islander Peoples and communities: Guidelines for researchers and stakeholders. Canberra: Commonwealth of Australia; 2018.

2. National Health and Medical Research Council. Ethical conduct in research with Aboriginal and Torres Strait Islander Peoples and communities: Guidelines for researchers and stakeholders. Canberra: Commonwealth of Australia; 2018.

3. Ungunmerr MR. Dadirri: inner deep listening and quiet still awareness. A reflection by Miriam-Rose Ungunmerr. In: Foundation MR, editor. 1988.

4. National Agreement on Closing the Gap. The Australian Government,; 2020.

5. The New South Wales Ministry of Health. NSW Aboriginal Health Plan 2013-2023. North Sydney, Australia 2012.
